# Prolonged survival after surgical resection of cerebral metastasis from melanoma with multisystemic metastasis already present: a case report and literature review

**DOI:** 10.1590/1516-3180.2017.0003090317

**Published:** 2017-08-21

**Authors:** Samer Saad Hoz, Ahmed Aman Alkhaleeli, Awfa Aktham

**Affiliations:** I Neurological Surgery Residents, Department of Neurosurgery, Neurosurgery Teaching Hospital, Baghdad, Iraq.; II Neurological Surgeon, Department of Neurosurgery, Neurosurgery Teaching Hospital, Baghdad, Iraq.

**Keywords:** Melanoma, Neoplasm metastasis, Glasgow coma scale

## Abstract

**CONTEXT:**

Malignant melanoma is the third most common cause of cerebral metastases after breast and lung cancer. Despite advances in therapeutic options, the prognosis for patients with cerebral metastases from melanoma remains poor, with a median survival time of six months after diagnosis.

**CASE REPORT:**

A 65-year-old woman was diagnosed with a malignant melanoma on the third toe of her left foot. The tumorous spot was excised surgically. However, the melanoma reappeared after one year and skin biopsy confirmed recurrence of malignant melanoma. Investigations showed metastasis to the left pelvic region, left lobe of the liver and right lobe of the lung. The patient then received chemotherapy. Subsequently, the patient was brought to the emergency department with an altered level of consciousness (Glasgow coma scale: 9) and hemiplegia on the right side of her body. Computed tomography scans of the brain revealed hemorrhagic lesions in the parieto-occipital lobes of the brain. Urgent surgical evacuation was done to remove the lesion, following which the patient showed improvement in her score on the Glasgow coma scale and a concomitant decrease in weakness. She was discharged from hospital with full consciousness. The patient died of acute renal failure 14 months after the brain surgery and approximately 4 years after the initial presentation of the case.

**CONCLUSION:**

This case outcome is rare and shows the effectiveness of surgery to treat cerebral metastasis from malignant melanoma in a situation with multisystem metastasis already present.

## INTRODUCTION

Melanoma, the cancer of melanocytes, i.e. the pigment-forming cells of the body, is the least common but most lethal of all skin cancers.^1^ The onset of the disease is marked by uncontrolled division of melanocytes between the epidermis and dermis, which is followed by radial invasive growth and vertical growth phases, respectively.^2^ The main causative agent of melanoma is exposure to ultraviolet (UV) radiation, particularly UV-B.

Treatment of melanoma is palliative in nature. A clinical diagnosis is required, followed by wide-margin excision of the tumor. Excisional biopsies may remove the tumor, but further surgery is often necessary to reduce the risk of recurrence. Complete surgical excision with adequate surgical margins and assessment for the presence of detectable metastatic disease, along with short and long-term follow-up, is the standard treatment protocol.^2^ Several treatment regimens including interferon therapy improve the prognosis but have severe side effects.

## CASE REPORT

The patient was a 65-year-old woman who developed a dark skin lesion on the third toe of her left foot in February 2013. This skin was excised surgically in December 2013 but unfortunately was not sent for biopsy, and the lesion recurred at the same location in February 2014. A skin biopsy now confirmed the presence of a Clark’s level II melanoma with surrounding hyperkeratosis and invasion of the epidermis and superficial dermis. Then, with further diagnostic workup, ultrasonography of the abdomen revealed normal liver, gall bladder, spleen, kidneys, urinary bladder, ovaries and pelvic lymph nodes. However, three lymph nodes in the left inguinal canal and a few in the right canal were enlarged, such that the largest one measured 35 × 29 × 17 mm. T1 and T2-weighted gadolinium-contrasted phase contrast magnetic resonance imaging (MRI) confirmed the results obtained from ultrasonography, thereby confirming the presence of metastasis to the inguinal lymph nodes.

A computed tomography (CT) scan of the chest on January 18, 2015, revealed left basal lung consolidation of heterogeneous density with spots of calcification, measuring 50 × 42 mm, which was suggestive of metastasis. Bilaterally enlarged axillary lymph nodes, measuring up to 13 × 6.5 mm, presence of fatty hilum and small hypodense lesions of 7 × 6 mm in the left lobe of the liver were also seen. MRI of the abdomen confirmed the presence of solitary, well-defined lesions in the left liver lobe with dimensions of 8 × 7 mm, which appeared hypo-intense in the T1-weighted and hyper-intense in the T2-weighted and STIR images, respectively.

The patient was administered chemotherapy comprising cisplatin, vinblastine and dacarbazine for three sessions starting at the end of January 2015, for three months, after which abdominal ultrasonography was performed to assess the status of the metastasis. This showed vascularized hypoechoic lesions in the left femoral region with a maximum size of 50 × 35 × 50 mm, thus indicating malignant lesions or lymphadenopathy. A pelvic MRI confirmed the presence of lymphadenopathy through detection of three enlarged lymph glands in the left femoral region.

This was followed by surgical removal of the diseased toe and resection of the inguinal lymph nodes in May 2015. Gross analysis on the amputated toe revealed ulceration up to the phalangeal bone, lymph node necrosis and hemorrhage. Histopathological analysis confirmed the presence of recurrent malignant melanoma of Clark’s level V with malignant melanocytes present down to the subcutaneous layer, a Breslow thickness of > 4.0 mm with stage T4b ulceration, and total effacement of the inguinal lymph nodes with pigmented malignant melanocytes. The disease was in stage T4N1bM0 of the TNM classification of the American Joint Committee on Cancer (AJCC), and in pathological stage IIIC.

A CT scan of the chest was performed in June 2015, and this revealed two well-defined hypodense pulmonary lesions of 8 × 7 mm and 7 × 6 mm, respectively, which were indicative of secondary metastasis. However, there was a reduction in the size of the primary lung lesion that had been observed prior to chemotherapy. Nonetheless, the chemotherapeutic regimen was continued for another three months with a reduced dose.

**Figure 1.**
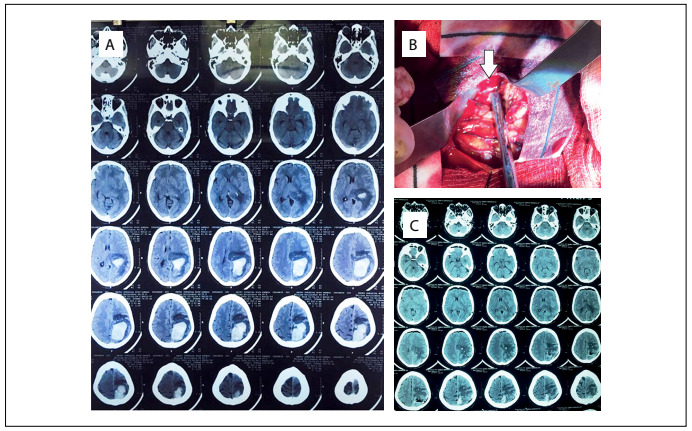
(A) Computed tomography (CT) scan images of the patient’s brain, showing metastatic hemorrhagic mass lesion before the cranial surgery. (B) Intraoperative photo showing the hemorrhagic mass (white arrow). (C) Immediate postoperative brain CT scan, showing removal of the lesion with relative reversal of mass effect.

On July 22, 2015, the patient suddenly complained of altered consciousness and hemiplegia on the right side of her body, with a score of 9 on the Glasgow coma scale (GCS). A CT scan revealed an intracerebral hemorrhagic mass, which was treated with urgent resection by means of parieto-occipital loop-assisted craniotomy on the same day (Figure 1A-C). Histopathological and cytological reports on the intracranial hematoma showed positivity for melan A and HMB-45. The patient was discharged after the surgery, on August 1, 2015, with improved consciousness (GCS 15).

Unfortunately, she died from renal failure on September 16, 2016, 14 months after the brain surgery and approximately 4 years after the first presentation of this case. This is a rare and unprecedented case among the among known occurrences of metastatic melanoma that were already multisystemic (Table 1).

**Table 1.** Diagnosis and treatment planning of the patient

2013 February	Black spot on left third toe.
2013 December	Excision of spot only without biopsy.
2014 February	Recurrence with biopsy done.
2015 January 18	Chest CT scan shows lung metastatic lesion.
2015 January	Start of chemotherapy: 3 sessions of 4 drugs, ending in March 2015.
2015 May	Left third toe excision + left inguinal lymph node resection.
2015 June	New chest CT scan shows a reduction in the size of the primary lung lesion that had been observed prior to chemotherapy.
2015 July 22	Sudden right-side weakness and disturbed consciousness, leading to urgent surgical evacuation by means of loop-assisted parieto-occipital craniotomy.
2015 August 1	Discharge with GCS 15, with improvement in weakness.
2016 September 16	Death due to renal failure.

CT = computed tomography; GCS = Glasgow coma scale.

## DISCUSSION

Several imaging techniques such as X-ray, ultrasonography, magnetic resonance imaging (MRI), CT scan and positron emission tomography (PET) scan are regularly used to detect metastatic melanomas.^3^^,^^4^ In the present case, combinations of all these methods were successfully used to evaluate the patient’s condition.

Here, we found that the patient presented a partial response to chemotherapy consisting of three drugs with anti-mitogenic effects. Despite complete remission in the case of the hepatic lesion and partial regression of the primary pulmonary lesion, the melanoma rapidly metastasized to the brain, thus indicating a requirement for invasive interventional methods.

Radiation therapy is another palliative method of choice that has been shown to extend life span by 3-5 months. It is often combined with systemic treatments such as use of immunotherapeutic drugs, to improve the prognosis of cerebral metastasis from melanoma.^5^^,^^6^ However, the precise location of the metastasis inside the brain (intra or extracranial) is a decisive factor for the extent to which the treatment can be used successfully with minimal side effects. Considering the drawbacks of the existing strategies, the search for better alternatives for treating brain metastasis from melanoma is still on (Table 2).

**Table 2.** Search of the literature in medical databases for case reports on melanoma conducted on May 2, 2017

Database	Search strategies	Papers found	Related papers
MEDLINE (via PubMed)	Radiation AND therapy AND lymph AND node AND dissection	2,163	2
MEDLINE (via PubMed)	Metastatic melanoma: chemotherapy review	1,706	3
MEDLINE (via PubMed)	Temozolomide melanoma	480	4
MEDLINE (via PubMed)	Interleukin-2 therapy melanoma	2,172	5
MEDLINE (via PubMed)	Intracranial metastases of malignant melanoma	193	6

Total number of articles retrieved from PubMed, removing duplications, was 5.

Here, we found that intracranial surgery significantly improved the clinical symptoms associated with brain metastasis from melanoma in our patient. The prognosis for brain metastasis from melanoma is poor, with a median survival span of 2-10 months.^3^^,^^4^

## CONCLUSIONS

This is the first report highlighting success from intracranial surgery to alleviate the symptoms of a patient with brain metastasis from melanoma that had progressed with systemic dissemination for a considerable length of time. This case is unprecedented in the history of prognoses from multisystemic metastatic melanoma. The outcome from this case is rare and shows the effectiveness of surgery in improving the prognosis and treating brain melanoma that already has systemic metastasis.
